# Progress in ambient assisted systems for independent living by the elderly

**DOI:** 10.1186/s40064-016-2272-8

**Published:** 2016-05-14

**Authors:** Riyad Al-Shaqi, Monjur Mourshed, Yacine Rezgui

**Affiliations:** Cardiff School of Engineering, Cardiff University, Queen’s Building, The Parade, Cardiff, CF24 3AA Wales, UK

**Keywords:** Ambient assisted living, Independent living, Smart homes, Elderly, Ageing, Dementia

## Abstract

One of the challenges of the ageing population in many countries is the efficient delivery of health and care services, which is further complicated by the increase in neurological conditions among the elderly due to rising life expectancy. Personal care of the elderly is of concern to their relatives, in case they are alone in their homes and unforeseen circumstances occur, affecting their wellbeing. The alternative; i.e. care in nursing homes or hospitals is costly and increases further if specialized care is mobilized to patients’ place of residence. Enabling technologies for independent living by the elderly such as the ambient assisted living systems (AALS) are seen as essential to enhancing care in a cost-effective manner. In light of significant advances in telecommunication, computing and sensor miniaturization, as well as the ubiquity of mobile and connected devices embodying the concept of the Internet of Things (IoT), end-to-end solutions for ambient assisted living have become a reality. The premise of such applications is the continuous and most often real-time monitoring of the environment and occupant behavior using an event-driven intelligent system, thereby providing a facility for monitoring and assessment, and triggering assistance as and when needed. As a growing area of research, it is essential to investigate the approaches for developing AALS in literature to identify current practices and directions for future research. This paper is, therefore, aimed at a comprehensive and critical review of the frameworks and sensor systems used in various ambient assisted living systems, as well as their objectives and relationships with care and clinical systems. Findings from our work suggest that most frameworks focused on activity monitoring for assessing immediate risks, while the opportunities for integrating environmental factors for analytics and decision-making, in particular for the long-term care were often overlooked. The potential for wearable devices and sensors, as well as distributed storage and access (e.g. cloud) are yet to be fully appreciated. There is a distinct lack of strong supporting clinical evidence from the implemented technologies. Socio-cultural aspects such as divergence among groups, acceptability and usability of AALS were also overlooked. Future systems need to look into the issues of privacy and cyber security.

## Background

The elderly population in the world is increasing as a result of the advancements in technology, public health, nutrition and medicine (Beard et al. 2012; Aytac et al. 1999). Rising life expectancy, declining birth rates and infant mortality will continue to influence this significant shift in demographics around the world, although at varying degree and pace (United Nations 2013). People aged sixty or over were more than 11.5 % of the global population in 2012. By 2050, this percentage is expected to double to two billion, and around thirty-three countries will have more than ten million people each, aged sixty or over (Haub 2012). The Organisation for Economic Co-operation and Development (2005) forecasted that during the first half of the twenty first century, its member countries would experience a drastic increase in the elderly population, as well as a steep decline in their working force population. For example, the percentage of the population aged 65 or over in the UK increased to 16 % of the total by 2009 while forecast suggests that 40 % of the country’s population will be aged fifty or over by 2026 (Winkler et al. 2007).

**Fig. 1:**
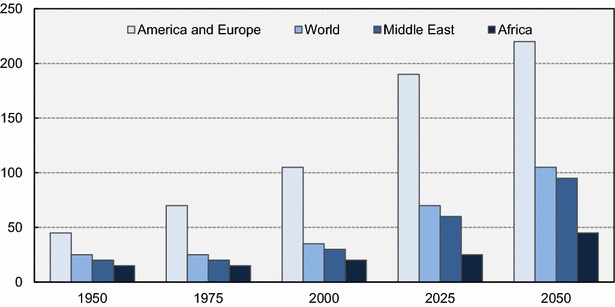
Number of persons aged 65 years or over, per hundred children under 15 years. *Data source*: (UN 2012)

The demographic shift is not evenly distributed. Figure 1 illustrates the number of persons aged 65 years or over, per hundred children under 15 years in different regions between 1950 and 2050. The ageing of the population in the developed America and Europe is steep compared to Africa. Ageing in the Middle East is expected to rise rapidly over the next 35 years. In Asia, the Chinese population is aging rapidly, due to the one-child policy that the government enforces and the country’s lower mortality rate (Zhang and Goza 2006). In the Middle East, the percentage of elderly to young people is low compared to western countries. However, the percentage of the aged population will increase throughout the region, with sharp increases in countries with declining fertility and extensive development. By 2050, around 22 % of the forecasted 1.1 billion people in the Middle East is expected to be of 60 years or over (United Nations Population Fund 2012). Although this share will still be low, the pace of aging will rapidly increase and by 2030 it is forecasted to be around 7 % (Hayutin 2009).

### Independent living by the elderly

Many older people want to spend time in their home environment. Nearly 40 % of the world’s elderly population live independently (United Nations 2012), almost half of whom are women while only a minority of older men live alone (Dwyer et al. 2000; Mba 2013). There are significant differences in the percentage of elderly people living independently in developed and developing countries. Elderly people who live independently represent around 75 % in developed countries (United Nations 2012). It is important to note that living alone or just with a spouse in developed countries may be regarded as economic independence while it may be an indication of vulnerability in developing countries (World Health Organization 2011), where social norm expects the older offspring to look after their parents in old-age.

A nationwide survey in the UK by the Disabled Living Foundation (DLF) (2009) revealed that more people worry about losing their independence (49 %) than dying (29 %) as they grow older. Similar results have been found in a survey in the USA by the Home Instead Senior Care, the largest elderly care organization in the world (Mangoni 2014). The DLF survey findings also suggest that losing independence or becoming dependent on others was a bigger concern than financial worries, despite the survey being conducted during a stringent financial times in 2009. The home is, therefore, a focal point for ensuring independent, healthy, and socially inclusive living, and should be designed and equipped with the right infrastructure to support and host a variety of services that older people may require to meet their needs (Shikder et al. 2010). Moreover, easy access to the social environments (e.g. healthcare facilities, care support, supermarkets, cultural centers and places to socialize) either from homes, or integrated into homes through adapted digital technologies, is essential to offset potential challenges such as isolation, loneliness, and associated physical and mental decline. Access to useful, high-quality information is also vital for making informed choices about elderly care, particularly for those suffering from gradual cognitive impairment. Studies have shown that most elderly people living with neurological conditions give priority to live independently in their homes, even though they may be dependent on others for the management of their daily life (Chan et al. 2009).

### Ambient assisted living

Ageing population will bring about some challenges for society (United Nations Population Fund 2012; Kwan 2012). Prolonged ageing has also resulted in an increase in neurological conditions such as age-related cognitive decline and chronic conditions among the elderly (World Health Organization 2006). Their quality of life is also affected by constraints related to physical activity, hearing and vision, and ultimately the loss of independence (Shikder et al. 2012). One of the key healthcare challenges is thus related to the provision of sustainable care to the growing number of elderly either in their homes or assisted living environments—by providing them with personalized care based on their profile and the surrounding context, commonly referred to as *ambient assisted living system* (AALS). Continuous and often real-time monitoring of the environment, and occupant behavior and health are the basis of AALS that provide a facility for triggering assistance through an event based system. These enabling technologies, along with preventative measures/care for healthy and active ageing are considered as the way forward from the perspectives of health and social care providers and professionals alike. The rationale is that healthy, active ageing can support independence—enabling the elderly to live well with simple or stable long-term conditions, as well as with complex co-morbidities, dementia and frailty (Oliver et al. 2014).

AALS provides user-specific support within the home environment, including the automated operation of equipment for *maintaining comfort* (e.g. heating, ventilation and air conditioning—HVAC systems), *safety* (e.g. lights) and *warning* (e.g. alarms for medicine) (Van Hoof et al. 2010). The system also enables support for tedious work; e.g. mobile and home robots offer assistance for moving objects or presenting food (Urdiales et al. 2013). For the elderly with cognitive impairments, the support for the tedious task is often *responsive*; i.e. the subject’s daily activities are monitored first to identify activities and then support is provided for the identified task. Some systems perform specific tasks that require interaction with outside agents or systems; e.g. paying bills, ordering groceries, etc. (van den Broek et al. 2010).

Significant advances in telecommunication; computing and sensor miniaturization; and the ubiquity of mobile and connected devices are influencing the development of AALS. Despite their recent progress and demonstration of positive effects on elderly people’s daily living (Bharucha et al. 2009), several limitations of the research and practice of ambient assisted systems have been identified. First, most studies lack satisfactory clinical evidence in support of the enhancement of quality of life achieved by introducing AALS, a concern shared by Blaschke et al. (2009) and Demiris and Hensel (2008). Second, the level of end-users’ acceptance of the technology regarding usability, eligibility of implementation, and ethical and privacy issues, is not explored in detail (Or and Karsh 2009). Third, the needs and demands of end-users such as the elderly and carers are not specifically addressed, and many projects are designed based on the assumption of researchers (Or and Karsh 2009; Chan et al. 2008). Fourth, Blaschke et al. (2009) points out that health and care workers responsible for the elderly have not always been well informed about the implemented AALS, in particular, the aspects that affect their work practices.

### Study contents

As a growing area of research, it is essential to investigate the approaches for developing AALS in literature to identify common practices, limitations and directions for future research. This paper is, therefore, aimed at a comprehensive and critical review of the frameworks and sensor systems used in various ambient assisted living systems, as well as their objectives and relationships with care and clinical systems.

## Methods

Published literature over the past 15 years in relevant electronic and non-electronic resources of peer-reviewed journal and scientific articles were searched to identify sources dealing directly with the support for independent living by the elderly, with a particular focus on ambient assisted living systems. The aim was to conduct a comprehensive assessment of the technology and classify recent developments so that the gaps can be identified, if any. Electronic resources related to the research topic keywords were searched through ScienceDirect, IEEE Xplore, Web of Science, PubMed and Google search engines, including Google Scholar. The keywords used were: *elderly people*, *daily activities*, *environmental monitoring*, *assisted living technology*, *smart homes*, *behavior monitoring*, *activity recognition*, and *distributed sensing* with both ‘OR’ and ‘AND’ connectives between search words. The search was cross-matched between the search keywords to cover possible combinations in all of the research databases. The obtained results were filtered into organizational websites, specialized books, and scientific articles. Abstracts of selected articles and books were then screened to identify potential literature directly related to the research topic. Moreover, a further search was conducted to follow potential authors’ literature related to the topic under consideration. The criteria for selecting and retaining highly linked articles for detailed review were:

- Coverage of the concept and philosophy of behavior recognition of the elderly;
- Coverage of the details of the monitoring system for environmental and vital signs, especially with the elderly as test subjects;
- Studies with significant contributions in smart home design and implementation;
- Studies that investigated the effectiveness of assisted living technologies;
- Studies covering detailed implementations and research projects related to independent living of the elderly; and
- Key review papers and reports from established authors and health organizations.

Finally, around 133 papers related to the research topic were retained for further investigation. Obtained results were clustered for structuring and organizing discussions, into groups, namely: *activity modelling techniques*; *personal and environmental sensing and monitoring system*; *home environment characteristics*; and *recent research projects* that addressed independent living by the elderly.

## Developments in ambient assisted living systems

**Fig. 2:**
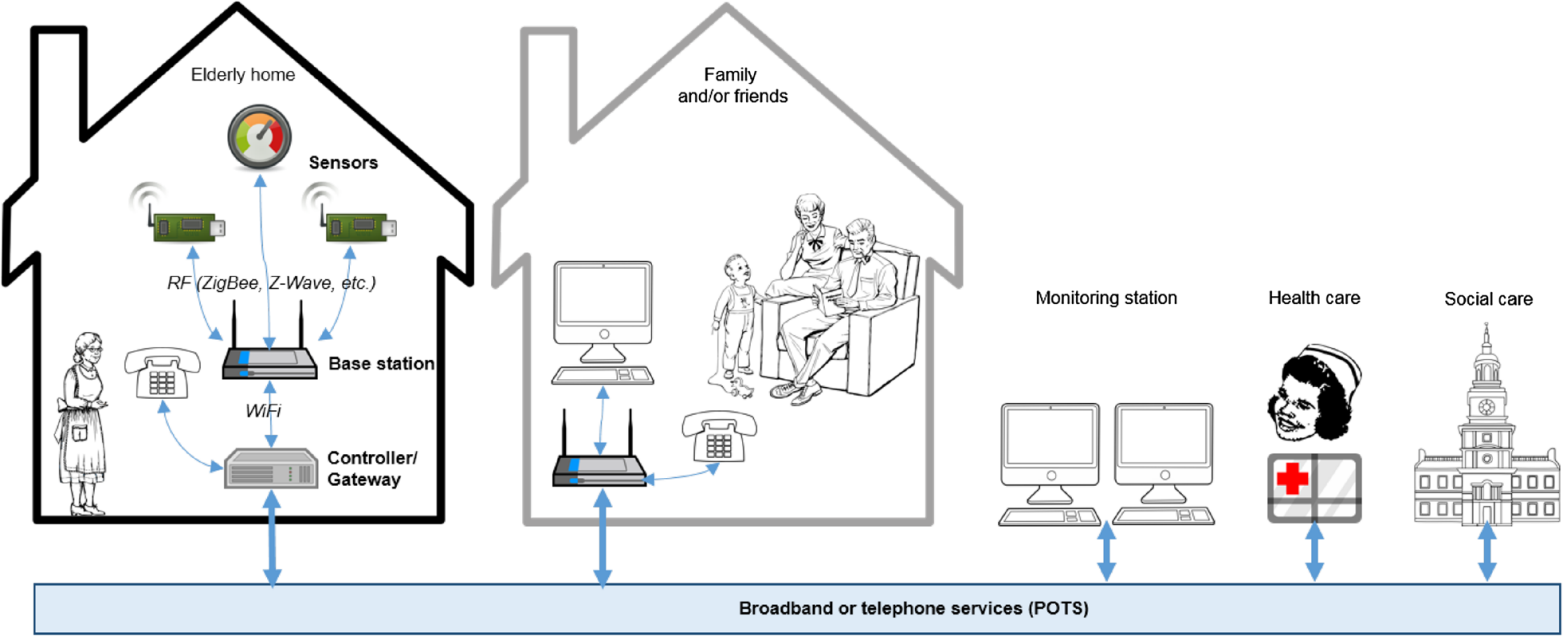
Architecture of a typical ambient assisted living system. Adapted from (Pollack 2005)

A typical AALS is illustrated in Fig. 2 where user behavior is monitored through a distributed home sensor system, which links caregiver and friends/family to the elderly’s home through an assurance system. In some applications, relatives and emergency services are also linked to the system for instant alerting in specific situations. Table 1 provides a list of commonly used sensor types in AALS, along with their usage, signal type, installation difficulty, generated noise and cost. Most sensors do not generate noise when operated and if not connected to alarms. Sensors that generate binary signals are typically easier to install and require less calibration than the continuous type.

**Table 1:** AALS sensor characteristics and cost

Sensor	Usage	Signal type	References	Device cost^a^ (US$)	Installation	Comment	Noise
Magnetic switch	Detect the opening of doors, windows, cabinets, etc.	Binary	Virone et al. (2008))	5.00 ± 0.75	Easy	High operational reliability. Maintenance free.	No
Temperature sensor	Detect ambient or water temperature	Continuous	Rowe et al. (2007), Virone et al. (2008)	9.00 ± 2.00	Moderately difficult	Normal operational reliability. May need frequent calibration.	No
Photosensor	Detect illuminance level	Continuous	Rowe et al. (2007), Muñoz et al. (2011)	5.00 ± 1.25	Moderately difficult	Location and orientation dependent	No
Pressure pad	Measure applied pressure at surfaces	Continuous	Virone et al. (2008), Muñoz et al. (2011)	25.00 ± 5.00	Difficult	Need frequent calibration.	Almost no noise
Water flow sensor	Measure flow in taps and showers	Continuous	Gaddam et al. (2010)	24.00 ± 3.00	Easy	Need frequent maintenance.	No
Infrared motion sensor	Detect motion or movement	Binary	Rowe et al. (2007), Virone et al. (2008), Muñoz et al. (2011)	35.00 ± 2.00	Moderately difficult	Normal operational reliability. May need frequent calibration.	No
Home electric appliances	Send signals when user turns equipment on/off	Binary	Rowe et al. (2007)	30.00 ± 5.00	Easy	Need frequent maintenance.	Almost no noise
Power/current sensor	Send numeric numbers according to electricity usage	Continuous	ONS (2008)	120.00 ± 3.00	Difficult	Needs professional installation and maintenance.	No
Force sensor	Detect movement and falls	Continuous	Kidd et al. (1999)	33.00 ± 5.00	Difficult	Need high adjustment	Yes
Smoke/heat sensor	Detect smoke or fire	Binary	Mitseva et al. (2009)	18.00 ± 6.00	Easy	Needs proper installation.	Yes
Biosensor	Monitoring human vital-signs	Continuous	Lie et al. (2011), Ichapurapu et al. (2009)	180.00 ± 5.00	Difficult	Need professional adjustment	No

^a^Indicative cost data, as of January 2015—collected by the authors from various US suppliers. Cost vary according to measurement accuracy, technology, device packaging and number of units

intelligence is gathered through a sensor network and fused together with data in which information and communication technologies and this equipment are introduced in order to assist inhabitants’ daily living activities such as moving furniture, timed medication, eating, dressing, communicating, and etc. The early stage of AAL homes projects focused on safety, in particular on alarm or notification in emergency situations such as the incidence of fall. and the system was generated primarily by users as listed in Table 2.

**Table 2:** Application, sensor location and aims of recent AALS projects

Project	Origin	Application			Sensor location		Monitoring aims			References
		Safety	Health and wellbeing	Social interaction	Physically fixed	Wearable	Environmental	Health	Cognitive	
AlarmNet	Virginia, USA	–	–	–	√	√	√	√	–	(Wood et al. 2008)
Assisted Cognition Environment	Washington, USA	√	√	–	√	√	√	√	√	(Qixin et al. 2006)
AWARE	Georgia, USA	√	√	–	√	√	√	√	√	(Kidd et al. 1999)
BioMOBIUS Research	Dublin, Ireland	√	√	–	√	√	√	√	–	(BioMobus 2011)
CASAS	Washington, USA	√	√	–	√	√	√	√	√	(Rashidi 2011, Cook et al. 2003)
Casattenta	Bologna, Italy	√	√	√	√	√	√	√	√	(Farella et al. 2010)
CodeBlue–Wireless Sensors for Medical Care	Harvard, USA	√	√	–	√	√	√	√	–	(Wood et al. 2008)
GatorTech Smart House	Florida, USA	√	–	√	√	–	√	–	–	(Helal et al. 2005)
Georgia-Tech Aware Smart Home	Georgia, USA	√	√	√	√	√	√	√	–	(Kientz et al. 2008)
Gerontological Smart Home Environment	Paris, France	√	√	√	√	√	√	√	–	(Gerontological 2011)
I-LivingTM	Illinois, USA	√	√	–	√	–	√	–	–	(Bal et al. 2011)
MavHome	Texas, USA	√	√	–	√	–	√	–	–	(Cook et al. 2003)
MIT House_n	Massachusetts, USA	√	√	–	√	–	√	–	–	(Chan et al. 2008)
ORCATECH	Oregon, USA	√	√	√	√	–	√	√	√	(Nehmer et al. 2006)
SISARL	Hsinchu, Taiwan	√	√	√	√	√	√	√	√	(Bal et al. 2011)
Smart Medical Home	New York, USA	√	√	√	√	√	√	√	√	(Ricquebourg et al. 2006)
SOPRANO	Patras, Greece	√	√	√	√	–	√	√	√	(Müller et al. 2008)
TAFETA	Ottawa, Canada	√	√	–	√	–	√	√	–	(Tafeta 2011)
WellAWARE	Virginia, USA	√	√	√	√	–	√	√	√	(Bal et al. 2011)
CareWatch	Scotland, UK	√	–	√	–	–	–	–	–	(Rowe et al. 2007)
TeleCARE	Scotland, UK	√	√	√	√	–	√	√	–	(Whitten et al. 1998)
CAALYX	Madrid, Spain	√	√	√	√	–	√	√	–	(Rocha et al. 2013)
Total	20	19	11	21	11	21	17	9		

It is evident from Table 2 that all projects (n = 22) primarily aim for proper environmental and subject condition monitoring, before going into achieving any required support function

The acronym, AALS, describes the ICT-augmented living environment in which ambient conditions are monitored via a sensor network and collected data are often fused together with information gathered from health and activity monitoring systems to (a) control the living environment for occupant safety and comfort, (b) provide family, friends and caregivers with up to date information on occupant status, and (c) inform short- and long-term health and care management. AALS is typically targeted at the elderly but the principles are similar to the paradigm of smart or intelligent homes, hence, it is applicable in a host of other relevant scenarios. Initial developments AALS centered around home automation in which distributed sensor systems were used to collect information about the state of the environment for performing certain actions and activating specific actuators to operate home devices and interchange data with outside domains.

The AAL home name was deduced from the main idea of home automation, which uses distributed sensor system to collect information related to the state of the environment where humans are located inside, then in response to this information decide certain actions and activate specific actuators to operate certain home devices, perform certain function, and interchange data with outside domains. AAL home may be also known as smart space, aware-house, and collaborative ambient intelligence. AAL homes that have these capabilities can deliver elderly people with various types of home assistance, controlled medication, fall prevention, security features, and etc. Such systems generate secure feeling for elderly inside home domain. Moreover, it will help relatives to observe their dear elderly from anywhere with an internet connection (Cheek et al. 2005).

Various laboratories trials, projects, and industrial showcases concerning AAL homes are available around the world; a lot of them share many features. Looking into the objectives these projects are aiming to achieve, they diverse in their technological innovation, information selection, validation method, and results confirmation.

In this respect, current technologies of AAL home could be categorized in three categories:

1. *Daily activities and social connectedness* Those targeting facilitating social activities, social networking, and identify social efficiencies.
2. *Safety enhancement* Those targeting fall detection, personal emergency, and medication management systems.
3. *Health monitoring* Those targeting managing chronic diseases. It also includes active tele-health allowing remote interaction with patient and collects continuous Health Records.

There are various projects worldwide, where some of which will be described in this section briefly.

### Daily activities and social connectedness

Assisted Cognition Environment (ACE) is aimed at the use of artificial intelligence (AI) techniques to enhance and provide support for daily life of the elderly suffering from cognitive by sensing the surrounding environment and the patient’s location, and interpret these data to identify behavioral patterns of the patient disorders (Kautz et al. 2002). Support is then offered to the patient through verbal and physical interventions, with the option to alert caregivers. Innovations in ACE can be divided into two: (a) the ability to create activity supervision model to reduce patients’ spatial disorientation, and (b) the structured prompter that supports patients in performing their everyday multi-step tasks (Qixin et al. 2006). On the other hand, AWARE project is aimed at conceptualizing the living context of the elderly by introducing ubiquitous computing to provide important information to their family members who are concerned about them living alone. The key innovation is the ability to distinguish a particular individual from others by detecting the person’s location using force sensitive load tiles on the floor that record foot step patterns, called ground reaction force (GFR), to create a model of unique footstep pattern of each individual. The GFR model is then compared with new GFR input data using hidden Markov models (HMMs) and feature-vector average (FVA) techniques to identify individuals. In addition, AWARE used radio frequency tags to locate frequently lost objects such as keys and glasses with a view to investigate, in a laboratory setting, how people lose their objects (Kidd et al. 1999). Successor to AWARE is the AWARE Smart Home project, which is aimed at improving social interactions between the elderly and their families, as well as the outside world (Kientz et al. 2008). Indoor position tracking was implemented using RFID sensors and computer vision based solutions to support an activity recognition system for identifying occupants’ activities; e.g. watching TV, reading and preparing a meal.

CASAS uses machine learning techniques to identify behavioral patterns of the elderly suffering from cognitive decline using data from motion sensors (Cook et al. 2003). Tests involving *cognitive healthy* and *dementia* subjects showed that the implemented learning algorithm was able to identify the differences in activities; however, it could not distinguish the cause of differences, whether it was a result of confusion due to dementia or a simple mistake. Managing an Intelligent Versatile Home (MavHome) project utilizes machine learning techniques to identify activities within a smart home environment, which is then used to actuate and control devices (home automation) with an overall aim to minimize the cost of maintaining the home and maximizing the comfort of its inhabitants (Cook et al. 2003). Lotfi et al. (2012) expanded CASAS by using a sequence of monitoring signals from different locations to describe the flow of occupant’s activity, alongside the duration of these signals. The collected data are analyzed using clustering techniques and have been found to be more effective in distinguishing abnormalities in activities by demented subjects. With similar objectives, I-LivingTM, on the other hand, focused on secure communication between distributed wireless sensors of different communication protocols (Bal et al. 2011), with an user interface designed to provide the differently-abled elderly to enhance their independence. The integration of several wireless network protocols (e.g. Wi-Fi, Infrared, Bluetooth, and IEEE 802.11) with commercially available sensing technologies for localization and presence identification was based on an open system architecture (Qixin et al. 2006). CAALYX is a European project focusing on three areas of monitoring in a social connected system: *home*, *roaming* and *central* care services (Rocha et al. 2013). The integration of wearable light devices such as data loggers in smart phones and watches sets CAALYX apart from other AALS in the sense that a larger set of parameters can be taken into consideration.

SISARL project focuses broadly on the use of consumer electronics to enhance the quality of life of elderly people and provide them with necessary help to achieve active and independent life (Bal et al. 2011). The project investigated several everyday living applications; e.g. the location of objects, use of medicine dispensers, monitoring of personal vital signs, detection of pattern irregularities, notifications, and the use of robotic platforms to enhance the dexterity and reachability of the elderly occupants. SOPRANO, a European project, is based on a combination of ontology-based techniques and a service-oriented device architecture (Müller et al. 2008). By separating system aspects such as sensors and actuators; context information and system behavior, SOPRANO provides a contract-centric framework for different solutions utilizing semantic technologies (Wolf et al. 2008). TeleCARE project presents a generic architecture for AALS (Whitten et al. 1998) through abstraction for both hardware and software, without specified information about dealing with third-party hardware drivers.

### Safety enhancement

Casattenta aimed at integrating ambient intelligence technologies, sensor fusion and wireless communication in the form of a set of fixed and wearable sensors distributed alongside the monitored environment connected through a communication platform. The system was designed to support independent living by enabling the tracking and identification of critical situations such as the danger of fall and immobility conditions (Farella et al. 2010). Gator-Tech was designed as an intelligent environment based on supportive features found in smart home devices such as smart appliances, plug-and-play sensors, actuators, and smart floors for position tracking. The overall system is based on a generic design for smart environment, containing the definitions of service for sensors and actuators distributed in the monitored environment, to support independent living by the elderly (Helal et al. 2005). CareWatch was developed for monitoring sleeping patterns of cognitive-declined elderly and activating notification systems for care providers, with a view to prevent unsupervised home exits to release some of the burden from the care providers, especially during the night. The system is designed to increase the quality of life for both the care recipient and the caregiver.

The Gerontological smart home environment (GERHOME) was intended to improve the feeling of independence for the elderly, complaining from the loss of autonomy (CSTB 2011). GERHOME implemented automatic recognition of human behaviors using real-time video surveillance combined with other types of sensor data. The project presented a communication infrastructure based on intelligent agents allowing easy integration of different types of sensors within an existing system structure, based on an intelligent agent architecture. Technology Assisted Friendly Environment for the Third Age (TAFETA) project built in the form of a smart apartment, loaded with various types of sensors and actuators to detect and control environmental parameters. The system was tested to monitor movement continuously in the apartment and to identify sleeping quality of occupants (TAFETA 2011). ORCATECH was devoted to the development of technologies supporting independent living for a wide range of requirements in elderly people’s health monitoring and home care support. The system comprises intelligent bed sensors to track sleeping patterns and prevent the elderly from falling by turning on room lights automatically when the system detects the person has awakened from sleep. ORCATECH also offers remote controlled tele-presence to provide health support to the elderly living alone, as well as enables social interactions with remote family members and caregivers (Nehmer et al. 2006).

### Health monitoring

BioMOBIUS project was developed as a research platform comprising hardware, sensors, software, services and a graphical development environment (BioMobus 2011), leveraging existing platforms and libraries such as EyesWeb XMI (eXtended Multimodal Interaction), conceived for supporting research and development on expressive interfaces and interactive systems for gesture recognition and movement analysis (Camurri et al. 2007). The platform implementation comprised a sensing infrastructure to monitor physiological parameters, a processing platform for data integration and fusion, and an intelligent agent that converted measurements into useful expressive information for the clinicians. The aim was to monitor blood pressure, gait stability, risk alertness, and social activity. The system was designed to be adaptable for various hardware through its generic mixed wired and wireless interfaces. The MIT House_n focused on the design elements and associated technologies of a smart home implemented in a laboratory facility equipped with sensors in various locations. The platform was designed to be extensible for further development of innovative user interfaces while investigating the needs for environmental conditions monitoring, proactive healthcare, biometric monitoring, indoor air quality, and new construction solutions needs for health and activity monitoring (Chan et al. 2008).

AlarmNet, a wireless sensor-based AALS, was aimed at providing health care monitoring for independent living (Center for Wireless Health 2011) by using heterogeneous wearable and stationary wireless sensing devices, which are combined with a user interface, database and decision logic. Mobile body worn sensors provide physiological sensing for blood pressure, pulse rate, and movement (accelerometer) data. Stationary sensors collect environmental data such as ambient temperature, air quality, light and user location. Collected data are filtered, aggregated, and analysed to adapt to residents’ requirements. AlarmNet’s flexibility allows the expansion of the system for integrating more sensing devices and for monitoring new parameters (Wood et al. 2008). Despite the intrinsic flexibility, AlarmNet is a closed architecture without the versatility of supporting third-party sensor and analytics. CodeBlue, on the other hand, was designed to examine the application of wireless sensor networks (WSN) for a range of medical applications including stroke patients’ rehabilitation and disaster response (CodeBlue 2011). The WSN comprised battery-powered sensor devices enhanced with enough computation and communication modules that collected and processed vital signs, which were then integrated into the patient care record system for real-time medical use (Wood et al. 2008).

Smart Medical Home focused on advancing interactive technologies for home health care (Ricquebourg et al. 2006). The project developed technologies to increase forward detection and anticipation of patient’s health and medical condition by using an interactive medical advisory system to interact with the patient. Using speech recognition and artificial intelligence techniques, together with patient’s available medical data, the interactive system advices residents for possible illness using structured interactive questions and answers in real time. WellAware provided an integrated structure comprising an unobtrusive sensor system and a user interface to enable professional caregivers as well as relatives to remotely monitor and deliver support to elderly people (Bal et al. 2011).

## Activity recognition

An activity recognition system typically consists of two sub-systems: (a) sensor system that is able to detect what happens in the environment, and (b) an intelligent model that is able to recognize activities from sensors information. The aim of ambient intelligence is to enrich the surrounding with modern sensor devices interconnected by a communication network to form an electronic servant, which senses changes in the surrounding, then reasons the causes of these changes, and selects proper actions to benefit users of the environment.

Direct sensing involves tracking the parameters that are related to the subject person himself, whereas indirect sensing focuses on identifying the environmental condition and spatial features. Both direct and indirect systems are employed in research and practice for capturing human behaviors. Direct sensing includes sound capture, video camera, and motion sensors, as well as wearable body sensors. Raw data/signals from these sensors are transferred to the database. Sensed data are typically annotated and often combined with each other to identify human behaviours in later stage of analysis. Health-related AAL systems can be divided into six main categories:

- *Physiological assessment* pulse rate, respiration, temperature, blood pressure, sugar level, bowel and bladder outputs, etc.
- *Functional assessment* general activity level measurements, motion, gait identification, meal intake, etc.
- *Safety monitoring* analysis of data that detect environmental hazards such as gas leakage. Safety assistance includes functions such as automatic operation of bathroom/corridor lights, reducing trips and falls.
- *Security monitoring* measurements that detect human threats such as intruder alarm systems and responses to identified threats.
- *Social interaction* contain video-based communication to support mediated connection with family and virtual participation in activities etc.
- *Cognitive monitoring systems* automatic reminders and other cognitive aids such as automated medication, key locators, etc. They also include verbal task instruction technologies for appliance operation and sensor assisted technologies that help users with deficits such as sight, hearing, and touch (Demiris and Hensel 2008).

Distributed computing enables wider deployment of technology in everyday life. Smart sensors, devices, and actuators have become more affordable, powerful and easy to install. Rapid developments in embedded systems and in particular the system on chip (SoC) low power computing architecture such as ARM SoC (Furber 2000) enabled the embedding of intelligence in everyday devices and equipment. Patients can now be observed and assisted their own home instead of mobilizing them to hospitals, resulting in economical and secure care supervision (Dengler et al. 2007). Feature rich smartphones can have bi-directional communication with cloud infrastructure to offload compute-heavy tasks, offering opportunities for rich functionalities. They can be used to attract elders’ attentions to certain actions, requirements, or guidance, while going about their daily activities, as well as communicate certain information to supporters and family members in critical situations. Consequently, these technologies can reduce healthcare costs significantly as well as the physical burden on health care supporters and family members (Fahim et al. 2012).

The challenges of AALS effect on users were investigated by Allameh et al. (2011) that identified that user’s acceptance of personal space modifications depends on user needs and lifestyle preferences. They worked classified the developments in AALS into three: ambient intelligent space (AmI-S), physical space (PS), and virtual space (VS)—integrated together to support independent life. Moreover, their model allows for changes in lifestyles due to changes in user activity. Currently, there is an interest for more detailed investigations on the linkage between AALS and user’s lifestyles.

Eunju et al. (2010) investigated the principles of activity recognition and demonstrated that it can be expanded to achieve increased societal ben-efits, especially in human-centric applications such as elderly care. Their application focused on recognizing simple human activities. Recognizing complex human activities is challenging and an active area of research. The nature of the problem; i.e. understanding human activities require an understanding of the activity profiles or patterns. Of various techniques, the first one is related to activity recognition based on an initial personalized model. Hence, a conceptual activity model should exist in the first step, which is then utilized to build a pervasive identification system (Chen and Nugent 2009). The second technique focuses on utilizing algorithms based on probability to generate a model for activity recognition (Wu and Huang 1999). Two of the most common methods used for this purpose are the Conditional Random Field (CRF) and the Hidden Markov Model (HMM) techniques.

Le et al. (2008) illustrated a method that enables activity recognition of elderly who lives alone. They studied the case of a subject living in a house, equipped with non-invasive presence sensors, to detect and assess her loss of autonomy by studying the degree of activities performed. In their work, they first detected the subject’s mobility states sequence in different allocations around the space. Then, from such states, they extracted descriptive rules to select activities that most influence the subject’s autonomy. Medjahed et al. (2009) illustrated an activity recognition system using fuzzy logic in home environments with the help of a set of physiological sensors such as cardiac frequency, posture, fall detection, sound, infrared, and state-change sensors. They validated their approach on a real environment and used this activity identification approach to build a model for anxiety, with increasing or decreasing confidence according to the state of each sensor used. They successfully embedded the characteristic of data provided from different sensors using fuzzy logic which allowed recognition of daily living activities for generic healthcare applications.

The work reported by Helmi and AlModarresi (2009) is a fuzzy system for pattern recognition that was utilized for activity modelling using tri-axial accelerometers. The accelerometers were utilized to detect and classify human motion into four categories: moving forward, upstairs, downstairs, and jumping movements. Their identification system depends on three different features: standard deviation, peak amplitude, and correlation between different axes and used as inputs to a fuzzy identification system. Fuzzy rules and input/output membership functions were defined from the experimental measurements. Their results supported that fuzzy inference system (FIS) outperform other types of classifiers.

Papamatthaiakis et al. (2010) used data mining techniques to build a smart system that is able to recognize human activities. They studied everyday indoor activities of a monitored subject. Their experimental results showed that for some activities, the recognition accuracy outperform other methods relying on data mining classifiers. They claim that this method is accurate enough for dynamic environments.

Zhu and Sheng (2011) illustrate a method for indoor activity identification that links the subject’s motion and position data together. They attached an inertia sensor that detects the orientation in three dimensions to the subject’s right thigh for motion data collection, and used an optical position system to get the subject’s location data. The optical positioning system can be replaced by any other location detection system. This combination maintained high identification accuracy while being less invasive. They utilized two neural networks to identify basic activities. First, Viterbi algorithm for finding the most likely sequence of hidden states (Zhu and Sheng 2011) was employed to recognize the activities from motion data only, forming coarse classification stage. Second, Bayes’ theorem was applied to update the recognized activities from motion data in the first stage. They built a mock apartment to conduct their experiments. The obtained results proved the method is effective and producing acceptable results for activity recognition.

Chen et al. (2012) conducted a comprehensive survey examining the development in sensor based activity identification systems. They presented a review of the major characteristics of video-based and sensor-based activity identification systems to highlight the strengths and weaknesses of these techniques and to compare between data- and vision-driven activity recognition techniques. They categorized the assisted living technologies into two categories based on the sensing method: direct (Muñoz et al. 2011) and indirect (Virone et al. 2008).

## Implementation challenges

A survey on intelligent techniques used to support the elderly was presented in Pollack (2005). Several challenges in implementing effective technologies to support the elderly population still exist, including the employment of Artificial Intelligence (AI) techniques for reasoning under uncertainty. Despite the number of applications of machine learning and natural language processing techniques, the consideration of uncertainty and thereby the accuracy of recognition has not been robustly dealt with. Moreover, additional challenges arise from the integration with sensor networks, privacy, security, human–machine interaction, and cognition impairment. For example, Dibley et al. (2012) illustrated a cost effective real-time distributed sensor system for environmental monitoring that integrates several types of devices, such as temperature, humidity, motion, light, and magnetic sensors. They used ontology-based framework for pattern recognition, which eased the process of software development, as well as system integration.

### Data augmentation

Augmentation and annotation of data is necessary for effective data analysis and elimination of irregularity and error for improved accuracy in a sensor network. There are several categories of data that can be augmented: temporal (e.g. date and time) and spatial (e.g. location) are the major categories of augmentation to the raw data (Blaya et al. 2009; Franco et al. 2010; Virone 2009). Other approaches include the development of ontology to include more detailed categorized information with a view to explain sensor event and context of occupants (Muñoz et al. 2011). Other contextual information such as messages and the design of the room can also be added (Rowe et al. 2007). The addition of more specific information on raw data is suggested as the key to increase accuracy of activity recognition (Van Kasteren et al. 2010).

### Data transfer and communication

Signals from monitoring devices and sensors are illustrated as either binary values (ON or OFF) or continuous values (e.g. 21 °C in environmental temperature) to the activity monitoring system. There are several ways to transfer the signals to database. Once devices are activated, the signal would be transferred to local data storage such as personal computer through wired or wireless communication. The signals (raw data) might be annotated with information such as time of activation and location of monitoring devices. Systems based on structured communication cabling between devices, sensors and computers are important for reliable system performance. However, plug&play wireless systems can provide alternative communication means. Generally, integration with basic home services still can be fully achieved by structured cabling. Moreover, modern buildings have extremely poor radio transmission as many wireless devices already operating in the environment (Linskell 2011).

The study of Jara et al. (2013), clarified that Ambient Assisted Living (AAL) technology developers are interested in real-time wireless transmission of human vital signs for the purpose of personalized healthcare applications for elderly people. Currently, personalized healthcare is bounded by the subject’s vital signs availability, which is continuously changing. Hence, continuous monitoring of subject’s vital signs is essential to provide certain health condition assessment. Hence, such continuous vital signs monitoring requires integration of wireless communication capabilities and embedded processing systems into lightweight, wearable, portable, and reliable monitoring devices that can be attached easily to the subject. Moreover, an interactive user interface system is also needed that is easy enough to be used by both the subject and supporter. In their work, they proposed a Near Field Communication (NFC) protocol as the medium for personalized healthcare following the concept of Internet of things. NFC is a technology that can be easily integrated in smartphones and portable devices that possess identification capability and able to construct communication channels among them. NFC still has challenges regarding its performance, efficiency, reliability of data transmission, as a result of the constrained resources and latency. These challenges are inherent to NFC technology as it is originally designed for simple identifications purposes, not for continuous data communication and processing as required for personalized healthcare. Hence, their work main novelty lies in designing a set of continuous vital signs data transmission devices communicating based on an optimized NFC system. Their system was integrated with user interface applications to provide information for caregivers and patients to support monitoring and managing patient’s health status using wireless communication. In their work, they also performed a technical assessment on the system latency and usability regarding their NFC communication system usage for continuous vital signs monitoring, through performing practical implementation of the system on a group of elderly people and their caregivers.

In a recent study by Arai (2013), he demonstrated a system that is able to continuously monitor subject’s health condition. Moreover, he proposed a correction algorithm to eliminate errors in physical health monitoring introduced by wearable devices. All types of wearable sensors monitoring body temperature, pulse rate, blood pressure, number of steps, calorie consumption, accelerometer, EEG, and GPS information, are considered in this study. Monitoring data were transferred from patient’s wearable devices to patient attached mobile device via Bluetooth. The mobile device is connected to the Internet through wireless communication network. Hence, the vital signs and psychological health data can be directly transmitted to the Information Collection Centre (ICC) for the purpose of health condition monitoring or help from designated caregivers when needed.

**Fig. 3:**
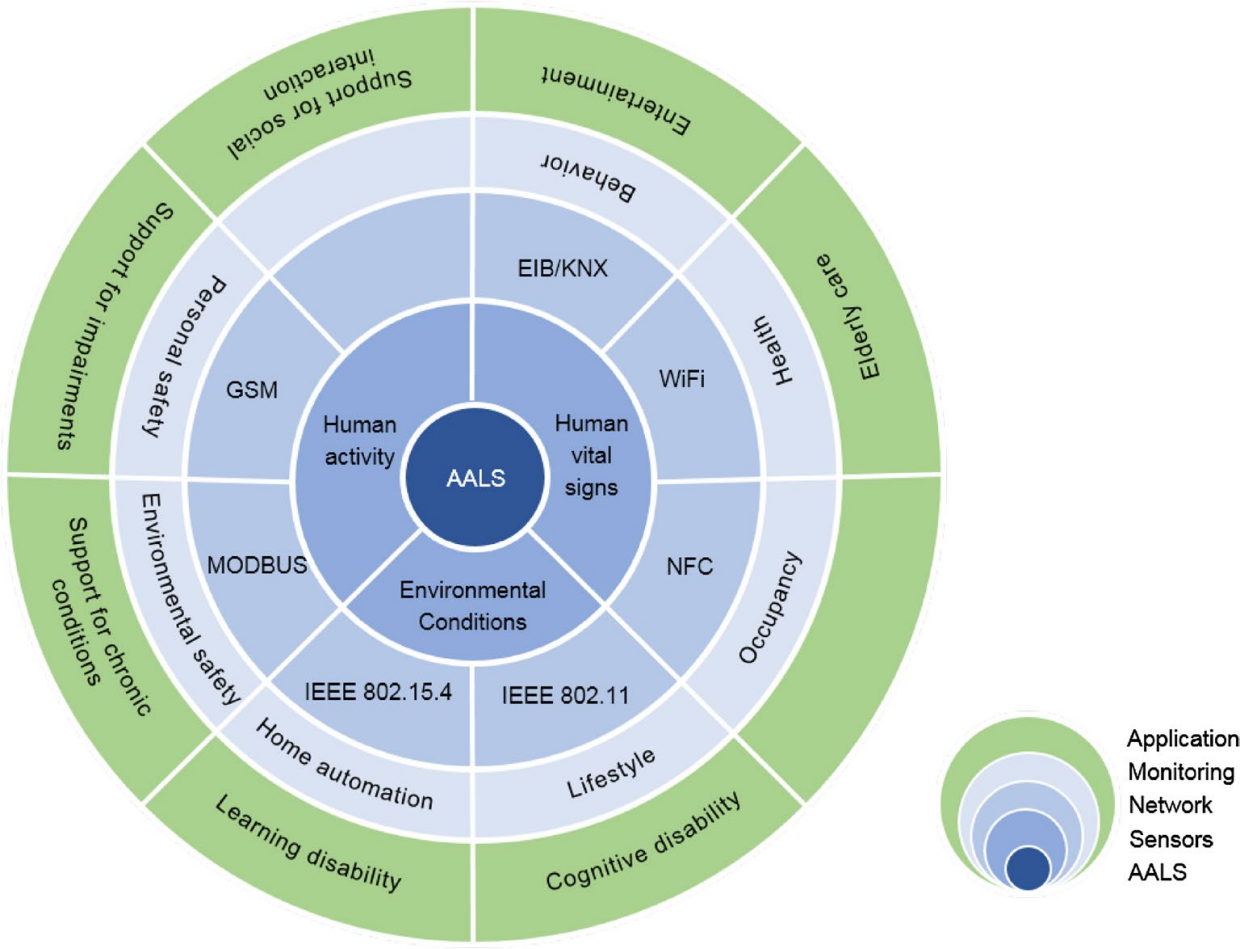
AAL system for providing different type of support

From previous information, it can be seen that technologies has be demonstrated in many ways to form a closed system able to provide specific types of support. Within UK, many categories of AALS have been developed to address specific needs or target group with specific technology implementation. Figure 3 illustrated below, summaries most of the activities illustrated in UK in the form of target group, provided support, and technology demonstrated in the AALS environment (Linskell 2011).

Sensor systems design is the first step in step for assisted living technology design. The second step is related to sensor data fusion for the purpose of activity recognition.

### Sensor fusion for activity recognition

Sayuti et al. (2014) discussed the trade-offs between measurements delay and throughput in a case study utilizing the lightweight priority scheduling scheme for activity monitoring from a distributed sensor system. The findings showed that the proposed scheme presented promising solution that supports decision making for Ambient Assistive Living (AAL) system in a real setting. The creation of an AAL environment not only embeds sensors to acquire information, it processes this information and interacts with the subject for enhanced quality of life.

There are several types of monitors which can be used to gather data of physical activities. In medical practice, it is common to continuously monitor patients’ biological status such as heart rates and saturation level by using wearable monitors. However, as some researchers have suggested, it is not practical to apply these wearable monitors in the community setting in order to evaluate their daily activities. Many remote monitoring projects have been developed in recent years in laboratory and community settings using non-wearable environment monitors. Xin and Herzog (2012) in their work presented a wearable monitoring system designed to achieve continuous in-house and outdoor health monitoring to support elderly people’s independence. The system acts as a health diagnosis assistant through its on-board intelligence to generate real-time reliable health condition diagnosis. The on-board decision support system continuously learns the subject’s health characteristics at certain time intervals from the attached sensor system. Hence, a dynamic decision model is continuously adapted to the subject’s health profile. The system is also able to measure deviations from the normal state and categorize whether it is a definite critical situation or a just a normal uncritical deviation.

Pirttikangas et al. (2006) studied activity identification using wearable, small-sized sensor devices. They attached these small devices to four different locations on the subject’s body. In their experiment, they collected data from 13 different subjects of both sexes performing 17 daily life activities. They extracted features from heart rate and tri-axial accelerometer sensors for different sampling times. They employed the forward-backward sequential search algorithm for important feature selection from these features.

De Miguel-Bilbao et al. (2013) illustrated a non-invasive sensor system that consists of action sensors and presence sensors for monitoring daily life activities, as well as the configuration of the monitored homes and users. The post processing stage for activity monitoring is independent from the home topology monitoring process. The system extracted parameters can be considered as long-term monitoring data aiming at detecting and validating daily activities, enabling early detection of physical and cognitive dysfunctions (Rashidi et al. 2011). The monitoring of household activity method can help to improve global geriatric evaluation and enhance the possibility for a better remote monitoring system of elderly people in their homes. This knowledge can support design and manufacture of biomedical sensors that are small, reliable, sensitive, and inexpensive (Agoulmine et al. 2011).

Shuai et al. (2010) focused on including activity duration into the learning of inhabitants’ daily living activities and behaviour patterns in a smart home environment. They applied a probabilistic learning algorithm to study multi-inhabitants in the same smart home environment. They predicted both inhabitants and their ADL model utilizing the activity carried out and the people who are performing it through experiments performed in a smart kitchen laboratory. The experimental results for activity identification demonstrated high accuracy compared to unreliable results that are obtained with no activity duration information in the model. Their approach also provides a great opportunity for identifying drifts in long-term activity monitoring as an early stage detection of deteriorating situation.

Language-based programming and interaction approach provides support for developers to freely express the global behaviour of a smart home application as one logical entity. The high-level language eases the implementation efforts for the application developer. By structuring the application development into different high-level models, developers can simplify application maintenance and customization due to changing user requirements or changes in the monitored living environment. In this way, people are directed to use rules for describing the required behaviour within a smart home environment. Consequently, by providing a rule-based modelling language the gap between the user-based application development and the actual system implementation can be reduced (Bischoff et al. 2007).

Algase et al. (2003) investigated reliable measures that are suitable enough to identify the wandering behaviour. Most of the studies they researched for wandering behaviour were relying on simple classification of subject’s state as wandering or not-wandering based upon personal caregiver judgments, which doesn’t have clear consistent assessment. They found that unplanned ambulation is a key element across all methods used for wandering behaviour identification. They studied different types of sensors used to wandering behaviour identification. They found that the StepWatch device outperform all other devices as it is always able to identify wandering behaviour correctly. The StepWatch always produced the best estimate for the subject’s wandering time spent, whereas other tested devices in the study were oversensitive to normal movement and produced substantial overestimates.

Wireless body attached sensor devices and smart phones were utilized to monitor the health condition of elderly people in a recent study by Bose (2013). These body attached devices offered remote sensing for the elderly vital signs for health condition assessment anytime and anywhere. Moreover, it supported creating customized solution for each subject according to his individual health condition requirements. If the system detects an emergency situation or deteriorating conditions, the smart phone will alert pre-assigned supervisors or the elderly person’s family or neighbours through text messages or making a phone call with predefined condition description voice message. In some cases, it even alerts the ambulance service with detailed report for the subject condition and location. Moreover, the system features some unique functions to support the elderly person’s daily life basic requirements, such as regular medication reminder, medical guidance, etc. However, he highlighted in his work that there is still need for innovations required in the Wireless Sensor Networks (WSN) field to enable such technologies to reach reliable and confidence application in this domain.

The work illustrated by Arai (2014), concerned with vital signs monitoring such as blood pressure, body temperature, pulse rate, bless, location/attitude, and consciousness using wearable distributed sensor network for the purpose of rescue of elderly people who will be in vital need for support in evacuation condition from a disaster location. Experimental results show that all of the vital signs as well as location and attitude identification of the elderly persons were correctly monitored with the proposed sensor networks. Moreover, it was clear that there is no specific correlation between pulse rate and the subject age, there is no specific calorie consumption that can be linked to age, EEG signal can be linked to eyes movement to predict psychological state, and there is clear difference between healthy person and patient with dementia disease. Finally, they found that there are links between blood pressure and physical/psychological stress (Arai 2014).

Phua et al. (2009) illustrated studied memory and problem-solving abilities to produce what then they called Erroneous-Plan Recognition (EPR), aiming to identify imperfections or faults in specific plans implementation by memory problems’ patients. Several challenges faced the researchers that are related to the correct definition of a plan within daily living activities, the choice of the activities to be monitored, the type of sensors required to recognize these activities, and the activities recognition technique to be used. In this study, they used independent sequential error detection layers to identify specific errors in the plan implementation. Their obtained result indicated that error data can be separated effectively. This study gave examples of how the suggested EPR system can work well with Deterministic Finite-State Automata (DFSA) technique for identifying error probabilities.

Lauriks et al. (2007) provided detailed analysis for the state of the art in information and communication technologies (ICT) that can be applied in solving unmet needs by elderly people. They categorized these needs as tailored information system requirement, customized disease support requirement, social interaction requirement, health condition monitoring requirement, and observed safety requirement. ICT solutions targeting memory problems demonstrate that people with memory diseases are able to use simple electronic equipment with enough confidence. Instrumental ICT-based systems targeting social activities could be simply implemented via the use of mobile phones or entertaining robotic platforms. GPS-based tracking devices proved their ability to enhance feeling of safety. However, more studies regarding these ICT solutions in simulated daily life situations are required before going to commercialized implementation for elderly people daily life support. The final step after sensor data fusion is the activity recognition algorithms used to characterize the activities performed by the elderly.

### Activity recognition mechanisms

#### Application of probability theory

The datasets in the database must be statistically analysed by probability theory and regression analysis which will show some trends within the datasets. To eliminate noise from raw data and to detect patterns, probability distribution and cluster analysis must be employed in which annotated information must be the key for efficient data cleaning. The analysis method must be modified depends on the type of data such as binary or continuous. Based on the patterns of the data, behavioural models/algorithms could be constructed which can be used in machine learning or fuzzy decision making systems. Once behavioural mode has established new input data will be compared with the model/algorithm inside the computer and will be evaluated as “normal or abnormal” which will represent the activity of inhabitant in the environment. In some cases, identifying unusual day consists of irregular patterns and rhythms of behaviours through pattern mining using templates of behaviours based on current/last day activities and circadian activity rhythm (CAR) (Virone et al. 2008; Junker et al. 2008). Hence, it is essential to recognise activities from various sets of sensors data into usable information. Consequently, AAL simulators need realistic sensors data.

The study presented by Chikhaoui et al. (2012) illustrated an autonomous system for activity identification in a controlled environment linking the activities and extracted patterns from sensor data together. They used pattern mining techniques linked with probability theory to discover and recognize activities. In their work, they presented activity recognition system as an optimization problem in which activities are modelled as probability distributions over sequential patterns. The experimental results were extracted from real sensor data placed in an AAL environment. Their results demonstrated the effectiveness of the suggested system for activity identification.

#### Application of wearable systems

Helal et al. (2012) illustrated an automatic situation generation methodology to create faithful sensors system to monitor activities. Their system constitutes a 3D graphical user interface to achieve virtual spatial projection from simulated sensors network in virtual reality environment. This system gives users simulation data to contribute to activity recognition directly linked to a certain space. Their work showed how a 3D simulator named Persim, can be used for activities identification purposes in a virtual reality domain to fuse the datasets needed for real-time activity recognition application. Their system is structured based on computer interface used for generating data regarding activities carried-out by a virtual character in a virtual space using Persim 3D’s intuitive graphical user interface (Helal et al. 2011).

On the study of Lara et al. (2012) a system called Centinela was illustrated. This system combines the subject’s body acceleration measurements with his vital signs to produce high accurate activity identification system. The system targeted five main activities namely, walking, sitting, running, descending, and ascending stairs. Their proposed design consists of an unobtrusive portable detecting device and a mobile phone. After testing three different time window sizes and eight different classifiers, results showed that Centinela platform can achieve around 95 % accuracy, which outperform other techniques when tested under the conditions. Moreover, the results indicated that vital signs measurements are important to differentiate between different types of activities. This finding strengthens the claim that vital signs mixed with motion information, form effective method to recognize human activities in general better than depending on motion data only. The position of the sensor was an important point in the study, where scientists identified that locating the motion sensor at the chest of the elderly person eliminates conflicts that may come if attached to the wrist (Tzu-Ping et al. 2009). In addition to activity recognition, the system presented a real-time vital signs monitoring interface adding easy health conditions monitoring to activity recognition target.

Krishnan and Cook (2014) developed a wireless and nonintrusive sensor system that is able to capture the necessary activity information from sequence of sensor system measurements. In this study they proposed and evaluated a sliding time window approach to identify activities in a flowing fashion. To differentiate between different activities, they incorporated the so called time decay correlation weighting of sensor measurements within a time window. They concluded from their experiment that combining joint information of weighted current sensor measurements and previous contextual information generates best performing streaming activity identification system.

Chernbumroong et al. (2013) addressed the issue of developing an activity identification system for assisted living technology application from the point of view of user acceptance, personal privacy, and system cost. The main aim of the research study was to design an activity identification system for recognition of nine different daily life activities of an elderly subject taking into account these aspects. The study proposed an activity recognition system for an elderly person using non-intrusive, low-cost, and wrist worn sensor devices. Their experimental findings showed that their system can achieve classification accuracy that exceeds of 90 %. They performed further statistical tests to support this claim, were they proved that by combining measurement data from accelerometer with temperature sensor reading, activity classification accuracy can be significantly improved.

#### Application of motion systems

In another study presented by Dinh and Struck (2009) a fall detection system that is able to monitor elderly people’s daily activities for support in the case of emergency was presented. The fall detection function was performed using only one triaxial accelerometer device. The motion measurements were used as the inputs for a fuzzy logic inference system followed by a neural network that classifies the orientation of the subject. In case the basic stable position conditions are changed, the system could be modified easily through the fuzzy inference system membership functions and rules. The obtained results indicated that only one triaxial accelerometer is good enough to form a robust fall detection system, where knowledge-based identification technique presents an effective replacement to standard pattern identification methods in this application.

In another study by Xu et al. (2012) a sensing cushion was illustrated which collects information about personal seating postures to support alerting signal generation in case of sitting for a long time that affects health. The cushion’s is formed by two parts, a seat pan and backrest surface that are equipped with distributed pressure sensors, where pressure distribution data are collected by a local microcontroller, then transmitted wirelessly to a personal computer via a Bluetooth channel. The presented identification system was able to recognize nine different seating postures with very high accuracy to support advice about proper seating orientation over a long time of sitting.

Virone (2009) illustrated a pattern recognition system for assessing behavioural rhythms in assistive aging technologies. The method was evaluated in assisted living environment using motion sensors to establish motion-based behaviours of elderly based on their activity displacements’ habits. The method was extended to study specific patterns of everyday living activities assuming that activities could be pre-identified and adapted on the long term using activity learning system. The system was successful in detecting behaviours emerging from patterns of movements elaborated from motion sensors. However, the method feasibility was tested using semi-artificial data.

Dalton and OLaighin (2012) studied the performance of two different classifiers for physical activity recognition, a base-level and meta-level classifiers. They utilized different wireless kinematic sensors dedicated to each individual in a group of twenty five subjects performing certain fundamental physical activities inside a monitored environment. Participants were asked to perform these specific physical activities randomly in the environment. They extracted features from sensors measurements based on frequency-domain and time-domain analysis such as average magnitude, zero-crossing rate, auto-correlation, cross-correlation, central moments, spectral entropy, and dominant frequency. Then they used wrapper subset evaluation technique to reduce the obtained features vector size for classifiers comparison purposes. The essential finding from this study was related to the importance of the wrist and ankle sensors devices in physical activities recognition applications.

Junker et al. (2008) illustrated a method for identifying sporadically occurring gesture information from constant data streaming collected from body-attached motion sensors. Their method was based on partitioning continuous sensor data signals in a two-stage identification approach for gesture recognition. In the first stage, similarity search technique is employed to select data sections that contain specific useful motions information. In the second stage, these signals are classified for gesture recognition purposes using hidden Markov models. They claimed that this technique presents solid strategy for identifying various gesture orientations from motion sensors as illustrated from two different test cases in their study.

#### Application of vision systems

On the other hand, image processing has been used extensively for activity recognition in computer vision systems. Although its popularity, its application in real life scenarios was limited as it is not entirely automated and requires high computational resources for information processing. In some works, automatic video sequence segmentation is applied for activity spotting. These segmented parts of the video are passed to an activity recognition algorithm. Activity detection is achieved by localizing video sequences in times that contain potential information and events. Motion detection combined with trajectory extraction is used for spotting important intervals. In this way, un-important parts of videos, such as motionless frames or long sequences with the same pattern are ignored. Generally, regions of interest (RoI) are defined when motion undergoes changes, where sample interest points are identified and tracked over time until activity ends, resulting in video sequence to be processed. This method is used to separate moving pixels from static ones through inter-illumination differences. Activity recognition can be then performed using K-means or Chi Square Kernel algorithms.

Nicquevert and Boujut (2013) used egocentric vision technology to capture the actions of subjects from their visual point of view using wearable camera sensors. They applied this paradigm to achieve activities monitoring for clinical evaluation for the impact of the disease on persons with dementia. The identification of patients’ position is the most important factor. Location estimation from distributed cameras at home is not sufficiently precise, due to the presence of others, where location estimation from egocentric video gives added value. The indoor environment is modelled by a set of known places and a 3D model that localize the subject from his vision camera. It combines interest points and the structure from camera vision to recognize the activity for analysis of the behaviour and the sequence of tasks executed by the subject. For this work, a dataset containing the eyes’ location of the person performing the motion is recorded in order to compare with the gaze coordinates of the people watching these videos, where two points per frame are recorded (30 images per second). The subjects were asked to execute specific activities such as “preparing a meal” using different items placed in front of them. In total 17 videos of 4 min each are recorded from different participants. The relation between Actors’ and Viewers’ saliency maps is based on that there exists a time shift around 500 ms between the beginning of the activity and the gaze fixation on the target. Hence, our given a saliency map of the Viewer, the prediction of the Actor’s saliency map is possible by simple time shift.

## Discussion

It is evident from the review that none of the presented projects provide solutions to all the aspects of AALS discussed in this paper. In most studies it is assumed that the system has been designed based on the belief that the behaviour of the inhabitants will be consistent from day to day and will have a general pattern. Behaviour models have been developed by deterministic models, probability analysis and other methods based on recorded observations from a few days to weeks. As one can reflect of his daily activities such as brushing own teeth, one will not behave exactly in the same order and duration as it was the day before and unlikely to happen the next day. One might brush his teeth longer with floss, change the tooth paste, or lines the mouth three times not four times. A research finds interesting trends within their datasets for making “simulation models” which consists of designed irregular patterns of daily activities that the designed irregular patterns are not performed as it should be by participants. Hence, many of the conducted research touched on the subject of support for the elderly only on the top surface and lacks deeper investigation on dynamic irregular pattern identification for activity recognition. Consequently, a set of challenges can be set for future research to effectively address such irregularity in behavioral models.

Besides health monitoring, one important aspect often ignored is to address the entertainment needs of these people, which is equally important for their well-being (Alm et al. 2009). Elderly can improve their quality of life through the support of entertainment and making their lives more enjoyable (Alm et al. 2007). It has been reported that multimedia enabled entertainment tools can promote effective treatment plan for the elderly with memory problems (Alm et al. 2009; Tamura et al. 2004). However, further study is required to obtain a scientific conclusion and prove it. Such studies also need to identify the requirements of an elderly entertainment support system from both the perspective of elderly and caregiver, which is a challenging task.

There are existing many literature that address elderly monitoring from different perspectives, such as providing robotic assistance (Montemerlo et al. 2002; Gross et al. 2011), supporting reminding service (Si et al. 2007), delivering information services (Fink et al. 1998; Chang et al. 2009), health monitoring system (Ohta et al. 2002; Gupta et al. 2007) and health smart home (Le et al. 2008). A few of the works only talk about entertainment for elderly people (Matsuyama et al. 2009; Tamura et al. 2004), however, these works do not address for example, the requirement of an entertainment support system which is claimed to have positive effect on elderly life, due to lack of information on recreational activities for elderly. Hence, there is a need to study everyday activities that include entertainment requirements for the elderly and provide a system architecture supporting these requirements in conjunction with everyday living activities support requirements. The following sub-sections illustrate in details the elderly demographic distribution, and the level of support required.

### Commercial challenges

There are many barriers to technology uptake in smart home environment, especially for elderly people with specific needs such as dementia or Alzheimer’s disease. A lack of suitable outcomes framework to validate the installation as well as managing the whole process for assessing, “prescribing” and delivering technological solutions to meet specific needs. Limited experience with tele-care technology initiatives has demonstrated that pilot projects do not necessarily lead to wide scale of technology application. There is a lack of commercial concerns to provide smart home solutions for people with special needs due to most of the skills required exist in academia and with others outside the commercial environment (Linskell 2011).

### Technological challenges

One major challenge in home assisted technology is related to continuous identification of the subject’s vital signs and health conditions via wearable devices (Chan et al. 2008, 2009). The challenge is basically related to the acceptability, durability, easiness, communication, and power requirements of these wearable devices. For instant, such devices need to be not only providing vital signs measurements, but also provide an assessment of the subject condition that is close to the doctor assessment when examining any patient. It needs also to be versatile in design with minimum weight, skin effect, and burden on the subject on his everyday life activities. Moreover, the power life of its battery and communication ability should be strong enough to be operated for days or weeks without the need for recharging. Additionally, it should be fault tolerant with high resistance to impact, heat, cold, and water. Combing all these requirements in the wearable devices is a high challenge factor for senor technology developers, that if achieved will boast the home assisted technology systems to further new dimension.

Moreover, standards that are related to specifying elements of assistive living technology are almost unavailable for the system developers. Consequently, adaptability of different system components from sensors, communication protocol, decision support, and subject interaction method or language, is not maintained and every system is linked only to the developer initiatives. Availability of such standards will help system designers to integrate efforts and provide the market with the necessary devices and systems to meet the subject defined requirements.

### Social challenges

Elderly people in general are often consciously aware of their privacy and possible intrusion. Acceptance of AALS by the elderly can, therefore, be challenging as the system may be perceived as intrusive. Most of the reviewed research appears to ignore and assume that users will accept the system in the way they design it. With limited available literature and surveys for user acceptance from the monitored subjects, this assumption is not always well regarded. Acceptability is culture dependent and will vary from one society to another. Gender and age have been found to influence people’s perception of space (Mourshed and Zhao 2012), which may also affect the acceptability of a system, in particular where behavior is continuously monitored. A significant challenge for system developers is, therefore, identifying the level of user acceptance.

## Conclusion

Ambient assisted living systems reviewed here were aimed supporting the elderly to live an independent life; help care givers, friends and family; and to avoid harm to the patients. Findings from our work suggest that most frameworks focused primarily on activity monitoring for assessing immediate risks, while the opportunities for integrating environmental factors for analytics and decision-making, in particular for the long term care were often overlooked.

The potential for wearable devices and sensors, as well as distributed storage and access (e.g. cloud) are yet to be fully appreciated. Advances in low cost embedded computing and miniaturization of electronics have the potential for significant future developments in the area. There is a distinct lack of strong supporting clinical evidence from the implemented technologies. Socio-cultural aspects such as divergence among groups, acceptability and usability of AALS were also overlooked. Future systems need to look into the issues of privacy and cyber security.
